# Childhood Takayasu arteritis: disease course and response to therapy

**DOI:** 10.1186/s13075-017-1452-4

**Published:** 2017-11-22

**Authors:** Florence A. Aeschlimann, Simon W. M. Eng, Shehla Sheikh, Ronald M. Laxer, Diane Hebert, Damien Noone, Marinka Twilt, Christian Pagnoux, Susanne M. Benseler, Rae S. M. Yeung

**Affiliations:** 1 Division of Rheumatology, Department of Pediatrics, The Hospital for Sick Children, University of Toronto, 555 University Avenue, Toronto, ON M5G 1X8 Canada; 2 Department of Immunology, University of Toronto, Toronto, ON Canada; 3 Department of Medicine, University of Toronto, Toronto, ON Canada; 4 Division of Nephrology, Department of Pediatrics, The Hospital for Sick Children, University of Toronto, Toronto, ON Canada; 5 Rheumatology, Department of Pediatrics, Alberta Children’s Hospital, University of Calgary, Calgary, AB Canada; 6 Vasculitis clinic, Division of Rheumatology, Mount Sinai Hospital, University of Toronto, Toronto, ON Canada

**Keywords:** Takayasu arteritis, Children, Biologic therapy, Vasculitis

## Abstract

**Background:**

Takayasu arteritis (TAK) is a large vessel vasculitis that rarely affects children. Data on childhood TAK are scarce. The aim of this study was to analyze the presenting features, course and outcome of children with TAK, compare efficacy of treatment regimens and identify high-risk factors for adverse outcome.

**Methods:**

A single-center cohort study of consecutive children fulfilling the EULAR/PRINTO/PReS criteria for childhood TAK between 1986 and 2015 was performed. Clinical phenotypes, laboratory markers, imaging features, disease course and treatment were documented. Disease activity was assessed using the Pediatric Vasculitis Disease Activity Score at each visit. Outcome: disease flare defined as new symptoms and/or increased inflammatory markers necessitating therapy escalation and/or new angiographic lesions, or death. Analysis: logistic regression tested relevant variables for flare. Kaplan-Meier analyses compared treatment regimens.

**Results:**

Twenty-seven children were included; 74% were female, median age at diagnosis was 12.4 years. Twenty-two (81%) children presented with active disease at diagnosis. Treatment regimens included corticosteroids alone (15%), corticosteroids plus methotrexate (37%), cyclophosphamide (19%), or a biologic agent (11%). Adverse outcomes were documented in 14/27 (52%) children: two (7%) died within 6 months of diagnosis, and 13 (48%) experienced disease flares. The 2-year flare-free survival was 80% with biologic treatments compared to 43% in non-biologic therapies (*p* = 0.03); at last follow-up, biologic therapies resulted in significantly higher rates of inactive disease (*p* = 0.02). No additional outcome predictor was identified.

**Conclusions:**

Childhood TAK carries a high disease burden; half of the children experienced flares and 7% died. Biologic therapies were associated with better control of disease activity.

## Background

Childhood Takayasu arteritis (TAK) is the most common large vessel vasculitis in children. It is characterized by intramural granulomatous inflammation of the aorta and its major branches [1, 2]. Vessel inflammation primarily causes wall thickening, stenosis and thrombus formation resulting in organ dysfunction secondary to ischemia [3]; however aneurysms and dissections can be seen [4]. Childhood TAK is a devastating disease with mortality rates as high as 35% [5–8]. The pathogenesis remains unclear; however, inflammation is a central feature. Cell-mediated immune mechanisms and their distinct pattern of secreted pro-inflammatory cytokines are associated with TAK [9].

Control of vascular inflammation and prevention of irreversible vessel injury and organ damage are the main treatment objectives in TAK. Treatment recommendations from adequately powered pediatric and adult trials are lacking [3, 10, 11]. Most available evidence on immunosuppressive agents and their efficacy is derived from adult observational cohorts [12–14]. Corticosteroids are currently the mainstay of treatment, but approximately half of all patients require additional immunosuppression [15]. Cyclophosphamide, methotrexate (MTX), azathioprine, mycophenolate mofetil (MMF) or leflunomide have been used with variable response [11–13, 16]. A recent study suggested high efficacy of biologic therapies in adult TAK patients with refractory disease course [17].

To date, it remains unclear how to best control disease activity and prevent organ damage in children with TAK. Efficacy and safety of distinct treatment regimens remain to be determined. Individual risk factors for disease progression and flares in childhood TAK are unknown. Therefore, the aims of the study were: 1) to describe the presenting clinical, laboratory and angiographic features of childhood TAK patients, 2) to explore and compare efficacy and safety of childhood TAK treatment regimens and 3) to attempt to identify risk factors for adverse patient outcome in childhood TAK.

## Methods

This single-center retrospective cohort study included all consecutive children ≤ 18 years of age at disease onset and diagnosed with childhood TAK according to the European League against Rheumatism (EULAR)/Pediatric Rheumatology International Trials Organization (PRINTO)/Pediatric Rheumatology European Society (PReS) final classification criteria for childhood Takayasu arteritis (Ankara 2008) between January 1986 and September 2015 [18]. Prior to 2009, children were classified based on the American College of Rheumatology criteria for vasculitis [19, 20]. Children were identified from the institutional database. Two independent reviewers collected all data using standardized forms at predefined time points including; at presentation, 6 months (after induction treatment), during flares, medication change, interventions, complications and last follow-up (FAA, SS). This study was performed according to the Declaration of Helsinki and approved by the institutional research ethics board (1000022123).

### Data collection: demographic features, clinical, laboratory and angiographic findings

Baseline demographic data including age at symptom onset and at diagnosis, gender, ethnicity and past medical history were recorded. Clinical characteristics included constitutional symptoms such as weight loss, malaise and fever, as well as signs and symptoms of organ involvement. Arterial hypertension was defined as blood pressure > 95th percentile for age. Blood pressure discrepancy was defined as a difference of ≥ 10 mmHg between limbs. Laboratory results included erythrocyte sedimentation rate (ESR), C-reactive protein (CRP), complete blood cell count, creatinine, von Willebrand factor antigen, antinuclear and antineutrophil cytoplasmic antibodies, proteinuria and hematuria. Tuberculosis testing results were recorded.

Vascular imaging studies included conventional angiography, magnetic resonance angiography (MRA), computed tomography angiography (CTA) and echocardiography studies. Location, type and characteristics of lesions such as stenosis (<50% lumen), narrowing, aneurysm, dilatation, dissection, vessel wall thickening and post-contrast enhancement were captured. Vascular imaging was documented when performed within 1 month of the predefined data collection time points.

### Treatment and complications

Current medication, vascular and surgical interventions were recorded. Disease and treatment-related complications included arterial dissection, treatment-related side effects and severe infections necessitating hospitalization.

### Disease activity and damage

Disease activity was assessed at each visit using the validated Pediatric Vasculitis Activity Score (PVAS) [21] and the Indian Takayasu Arteritis Activity Score (ITAS2010), which was recently validated in adult Indian TAK patients [22]. Disease damage was assessed using the Pediatric Vasculitis Damage Index (PVDI), a damage assessment tool modified from the adult Vasculitis Damage Index [23]. The PVAS and PVDI were systematically completed at each clinic visit as part of routine clinical practice at our institution since 2009. For patient visits before 2009, the PVAS and PVDI were retrospectively completed by two independent reviewers (FAA, SS); discrepancies were resolved by a third reviewer (SMB). The ITAS2010 was retrospectively assessed for all visits. All three scores assess the multisystem involvement in childhood TAK. PVAS and ITAS2010 measure disease activity including symptoms that newly occurred or worsened over the past 4 weeks or are persistent for less than 3 months. The PVAS is currently the only validated disease activity measurement tool in childhood vasculitis and routinely used for clinical research [21]; however, it may not be optimal for assessment of disease activity in pediatric large vessel vasculitis. The ITAS2010 has been specifically developed for assessment of disease activity in TAK patients [22], but has not been validated in children and is therefore not routinely used in childhood TAK. Given these specific features, both, the PVAS and ITAS2010 were used for assessment of disease activity. In contrast to the PVAS and ITAS2010, the PVDI assesses irreversible cumulative disease-related damage and only includes symptoms present for more than 3 months.

### Definitions

Active disease was defined as PVAS ≥ 1 and/or raised inflammatory markers and/or imaging evidence of active disease including new angiographic lesions, vessel wall inflammation by post-contrast enhancement and thickening. Inactive disease was defined by the concurrence of all three, a PVAS of zero and normal inflammatory markers and inactive disease on imaging. Disease flare was defined as occurrence of new TAK symptoms and/or increase of inflammatory markers necessitating altered treatment and/or new or worsening angiographic lesions. The duration of stable disease prior to disease flare was not further defined. Inactive disease was defined by the treating expert clinician.

Treatment response was defined as a ≥ 50% reduction in the PVAS at 6 months after treatment start.

### Outcome

Primary outcome was disease flare or death. Secondary outcomes included disease activity as defined above and disease damage as measured by PVDI at last follow-up.

### Analysis

Patients were analyzed in groups depending on type of induction therapy. Flare-free survival of biologic or non-biologic treatments was calculated using Kaplan-Meier methods. As each child could experience multiple flares, a time-to-event analysis was used. Therefore, the time origin for each treatment was considered the time when the child was started on treatment. Logistic regression was used to identify prognostic factors of adverse outcome. The following variables were studied as potential predictors: age, gender, PVAS, ITAS2010, ESR, platelet count and treatment category (biologic versus non-biologic treatment). The analysis was adjusted for the number of treatment episodes per patient. Variables with more than 20% missing values were excluded. Categorical variables were compared by Fisher’s exact test, odds ratios (OR) were calculated including the 95% confidence intervals (95%-CI). Continuous variables were reported as medians with interquartile range (IQR), categorical variables as percentages with frequencies. Analyses were conducted in Prism (GraphPad 6.0 g, San Diego, CA, USA) and R 3.12 (Vienna, Austria).

## Results

### Demographic features, clinical and laboratory findings

A total of 27 children with TAK (74% females) were included. Twenty-two children (81%) had evidence of active disease at diagnosis. The median age at diagnosis was 12.4 years (IQR 9.1–14.4). The median duration from symptom onset to diagnosis was 6 months (IQR 2.9–15.2). Median follow-up duration was 2.1 years (IQR 1.2–5.5). Demographic features are summarized in Table 1.

**Table 1:** Demographic features

Patient characteristics at baseline	Patients (*N* = 27)
Female (%)	20 (74)
Age at diagnosis, years	12.4 (9.1–14.4)
Duration symptom onset to diagnosis, months	6 (2.9–15.2)
Follow-up duration, years	2.1 (1.2–5.5)
Ethnicity (%)	
Caucasian	8 (30)
Asian	4 (15)
Black	3 (11)
Hispanic	2 (7)
East Indian/South Asian	2 (7)
Middle Eastern	2 (7)
Aboriginal/First Nation	2 (7)
Unknown	4 (15)

Values are presented as absolute numbers (%) or medians (IQR)

The most common symptoms at presentation were malaise (48%), headaches (33%) and weight loss (30%). Blood pressure discrepancy (67%), decreased or absent pulse (59%), arterial hypertension (56%) and bruits over large vessels (56%) were the most frequent findings on physical examination. The presenting clinical features are shown in Table 2.

**Table 2:** Presenting clinical and laboratory features

Presenting features		Patients (*N* = 27)
Symptoms:		
Constitutional:	Malaise	13 (48)
	Weight loss	8 (30)
	Fever (> 38.0 C)	5 (19)
	Lymphadenopathy	3 (11)
Cardiovascular:	Claudication of the extremities	6 (22)
Neurological:	Headache	9 (33)
	Dizziness	5 (19)
	Stroke/TIA	3 (11)
	Syncope	3 (11)
Gastrointestinal:	Chronic nausea/vomiting	5 (19)
	Nonspecific abdominal pain	4 (15)
Pulmonary:	Shortness of breath	4 (15)
	Chest pain	3 (11)
Musculoskeletal:	Back pain	5 (19)
Ocular:	Blurred vision/uveitis/retinal hemorrhage	4 (15)
Findings on clinical exam:		
	Blood pressure discrepancy	18 (67)
	Decreased or absent pulse	16 (59)
	Arterial hypertension	15 (56)
	Bruits over large arteries	15 (56)
Laboratory characteristics:		
	ESR, mm/h	35 (17–74)
	CRP, mg/dL	31.9 (5.3–67.6)
	Hemoglobin, g/L	115 (96.5–125.5)
	White blood cells, × 10^^^9/L	8.8 (6.9–12.4)
	Platelets, × 10^^^9/L	366 (256–543)
	vWF antigen, IU/mL	1.6 (1.1–2.0)
	Impaired renal function	2 (7)
	ANA	8/22 (36)
	ANCA	2/18 (11)

Clinical features present in > 10% of the patients with childhood TAK are listed. Impaired renal function was defined as a creatinine increase of > 30% of the upper normal limit at diagnosis. Values are presented as numbers (%) or medians (IQR)

*TIA* transient ischemic attack, *ESR* erythrocyte sedimentation rate, *CRP* C-reactive protein, *vWF antigen* von Willebrand factor antigen. *ANA* antinuclear antibody, *ANCA* antineutrophil cytoplasmic antibody

Three patients (11%) were diagnosed with tuberculosis at presentation of childhood TAK. Two children had been diagnosed with inflammatory bowel disease based on classic histopathology findings prior to the onset of TAK. At time of diagnosis, inflammatory markers were elevated in 21 children (78%). ESR was increased in 20/25 (80%), CRP in 14/19 children (74%). Laboratory results are presented in Table 2. The median PVAS at diagnosis was 10 (IQR 7–18), the median ITAS2010 14 (IQR 10–18).

### Angiographic features

**Fig. 1:**
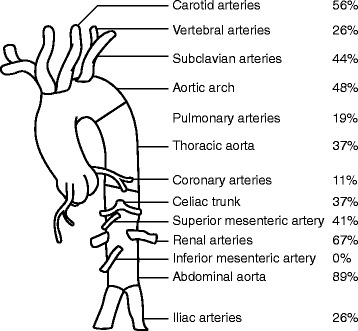
Frequency of arterial vessel involvement at diagnosis. Frequencies (%) of patients with any lesion (stenosis, narrowing, aneurysm, dilatation, dissection, vessel wall thickening and post-contrast enhancement) in the indicated vessel. Frequencies of paired vessels (*right/left*) are presented as one combined value

MRA alone (11/27, 41%) or in combination with CTA or conventional angiography (3/27, 11%) was most commonly performed at diagnosis. Conventional angiography was the initial imaging modality in 9/27 (30%) children, and CTA in 3/27 (11%). The most frequently involved vessels were the abdominal aorta (89%), the renal (67%) and carotid arteries (56%). Figure 1 depicts the frequencies of the involved vessels.

### Treatment

**Fig. 2:**
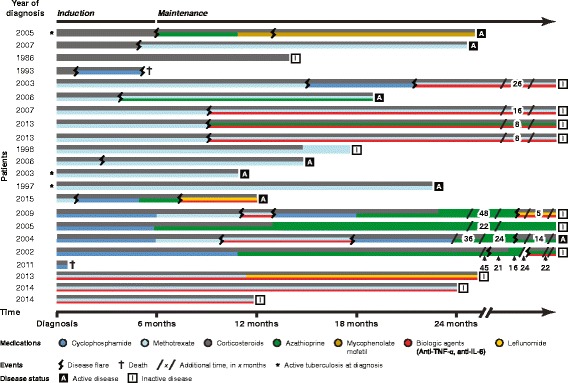
Induction and maintenance treatment regimens in children with childhood TAK presenting with active disease (N = 22). *Colored lines* depict different therapeutic agents. The patients are grouped according to their induction phase therapy: 1. corticosteroids (*grey*) only, 2. corticosteroids in combination with methotrexate (*light blue*), 3. corticosteroids in combination with cyclophosphamide (*dark blue*) and 4. corticosteroids in combination with biologic agents (*red*) and methotrexate. The timeline is drawn to scale up to 24 months; additional time of treatment is shown in months between break lines. Also depicted are clinical events, including disease flare, death, tuberculosis infection at diagnosis and disease activity (active/inactive) at last follow-up

**Fig. 3:**
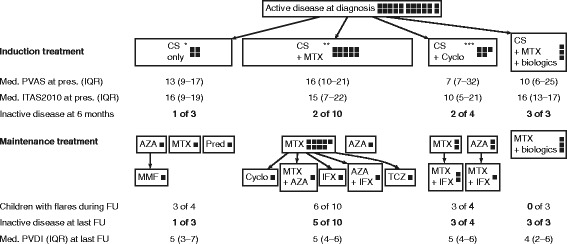
Measures of disease activity and damage in the 22 childhood TAK patients with active disease at diagnosis. The *squares* represent individual patients. *AZA* azathioprine, *CS* corticosteroids, *Cyclo* cyclophosphamide, *FU* follow-up, *IFX* infliximab, *ITAS2010* Indian Takayasu Arteritis Activity Score, *MMF* mycophenolate mofetil, *MTX* methotrexate, *PVAS* Pediatric Vasculitis Activity Score, *PVDI* Pediatric Vasculitis Damage Index, *TCZ* tocilizumab. *One child was started on cyclophosphamide treatment at 6 weeks and died at time of flare at 4 months after diagnosis. **One child was started on cyclophosphamide treatment 6 weeks after diagnosis. ***One child died 12 days after diagnosis

Twenty-two of 27 children (81%) received immunosuppressive therapy (Figs. 2 and 3). Five (19%) were considered to have inactive disease at diagnosis and therefore did not receive immunosuppressive agents.

### Induction treatment

Patients with active disease at diagnosis (22) received different treatment regimens. Initially, 4/27 children (15%) received high-dose corticosteroids only, and 18 (67%) received a combination of corticosteroids plus another immunosuppressive agent. These immunosuppressive agents prescribed in combination with corticosteroids included MTX in ten (37%), cyclophosphamide in five (19%), and MTX plus a biologic agent in three (11%) children. The latter three children received tumor necrosis factor alpha (TNF-α) inhibitors, two infliximab and one adalimumab. Two of them had been already on TNF-α inhibitors for pre-existing inflammatory bowel disease, when diagnosed with childhood TAK. At diagnosis of childhood TAK, both were started on high-dose corticosteroids and MTX; in addition, the TNF-α inhibitor dosing was increased. Therapy was chosen at the discretion of the treating physician and the availability of the drug.

PVAS and ITAS2010 did not significantly differ between the four induction treatment groups at diagnosis. At 6 months 20/22 treated children survived and 18/20 surviving children (90%) had responded to the treatment (Fig. 3).

### Maintenance treatment

At 6-month follow-up, all 20 surviving children remained on corticosteroids at a median dose 0.4 mg/kg/day prednisone equivalent (IQR 0.4–0.7 mg/kg/day). Maintenance treatment varied and included MTX, azathioprine, MMF and leflunomide or one of them combined with biologics. An overview of the treatment regimens is shown in Figs. 2 and 3.

### Co-therapies

Most children (18/27, 67%) received antihypertensive drugs (median 1, 0–3). Low-dose acetylsalicylic acid was prescribed to 15/27 (56%) and anticoagulation to 6/27 (22%) children. A 6 to 9-month course of antituberculosis treatment was initiated simultaneously with immunosuppressive therapy in the three children diagnosed concomitantly with tuberculosis.

### Surgical and endovascular interventions

Eight of 27 patients (30%) required vascular surgery or intervention after diagnosis of childhood TAK. Eighteen procedures were performed: renal artery angioplasty (eight procedures in four patients), balloon dilatation of the aorta (four procedures in two patients) and axillary-femoral bypass, coronary bypass and embolectomy of the femoral artery in one patient each. Another child required a splenorenal shunt, local thrombolysis due to thrombosis and eventual nephrectomy. Two patients received surgical treatment prior to childhood TAK diagnosis (unilateral nephrectomy with subsequent renal revascularisation procedure and balloon dilatation of the aorta).

### Complications

Complications were divided into disease- and treatment-related complications. Arterial dissection was noted in three children (11%) at diagnosis; no new dissections were seen during follow-up. Cerebral infarction with consecutive craniectomy (due to increased intracranial pressure) and intestinal ischemia requiring intestinal resection were documented in one child each. Relevant treatment-associated side effects included Cushing syndrome in 21/22 children on corticosteroids, transaminitis defined as transaminase levels ≥ twice the upper limit of normal in four children and vertebral fractures in one child. Four severe infections in four children required hospitalization including one child treated with corticosteroids and MTX who developed candidemia. Three children treated with corticosteroids and cyclophosphamide were hospitalized for severe Epstein-Barr virus (EBV) infection, varicella zoster infection and bacterial sepsis following bowel ischemia.

## Outcomes

### Primary outcome

A total of 14/27 (52%) patients experienced adverse outcomes; 13 (48%) developed a disease flare and two children died (7%), one during a flare. The first flare occurred at a median of 9.0 months after diagnosis (IQR 3.9–12.1). Twenty-one flares were observed during the study period: 16 (76%) were diagnosed by new angiographic lesions, three (14%) by both new clinical symptoms and increase of acute phase reactants and two (10%) by increase of acute phase reactants only necessitating treatment alteration (start of infliximab in one, dose increase of corticosteroids and methotrexate in the other patient). The number of flares varied between one and three per patient. All but one child flared while receiving immunosuppressive therapy. Flares were treated by intensification of therapeutic regimens such as increase or restart of corticosteroids and/or start of an additional immunosuppressive drug such as cyclophosphamide, azathioprine or a biologic agent. Figure 2 depicts flares and individual treatment regimens.

When analyzing treatment episodes separately, 19 flares occurred during 44 non-biologic treatment episodes (43%) compared to only two flares during 12 biologic treatment episodes (17%) (*p* = 0.18; OR 3.80, 95% CI 0.81–18.59). Median corticosteroid dose at time of flare was 0.4 mg/kg/day (IQR 0.2–0.9) prednisone equivalent. None of the patients with inactive disease at time of diagnosis experienced an adverse outcome.

**Fig. 4:**
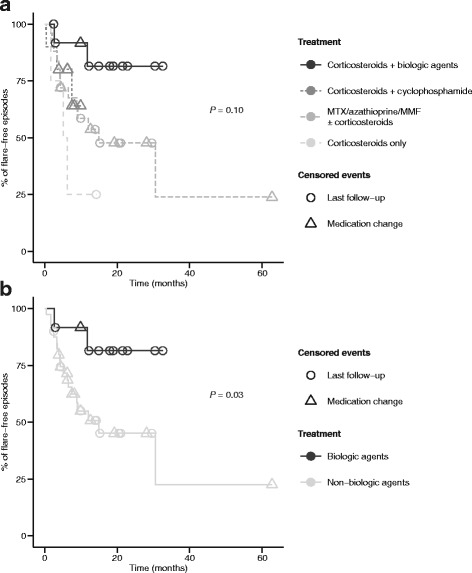
Flare-free survival in the 22 childhood TAK patients with active disease at diagnosis. **a** Flare-free survival separated into different treatment groups (biologics N = 12; cyclophosphamide N = 10; corticosteroids only N = 4; methotrexate/azathioprine/mycophenolate mofetil/leflunomide N = 30 treatment episodes), adjusted for the number of treatment episodes. **b** Flare-free survival grouped into biologic therapy (N = 12 treatment episodes) and non-biologic therapy (methotrexate, azathioprine, mycophenolate mofetil, leflunomide, corticosteroids, cyclophosphamide, N = 44 treatment episodes), adjusted for the number of treatment episodes

The 2-year flare-free survival was 80% with biologic treatments compared to 43% in non-biologic treatments when adjusted for the number of treatment episodes per patient (*p* = 0.03) (Fig. 4). Biologic agents included infliximab (5 mg/kg at 0, 2 and then every 4 weeks) in nine treatment episodes, adalimumab (40 mg every 2 weeks) in one and tocilizumab (8 mg/kg monthly) in two.

Two children died from TAK, both within 6 months of diagnosis. A four-year-old boy presented with a 3-month history of arterial hypertension, blood pressure discrepancy between limbs and absent pedal pulses. Conventional angiography revealed involvement of the abdominal aorta, the superior mesenteric, celiac and bilateral renal arteries. High-dose corticosteroids, anticoagulation and quintuple antihypertensive therapy were started. Cyclophosphamide was added 1 month after diagnosis due to progressive disease. He died 4 months after diagnosis following disease flare with uncontrollable hypertension and massive intestinal ischemia with sepsis. A 13-year-old girl presented with a 3-week history of constitutional symptoms, cough, chest pain, headaches and arterial hypertension. MRA showed extensive involvement of the abdominal aorta, celiac, superior mesenteric, pulmonary, renal and iliac arteries. She received high-dose corticosteroids, cyclophosphamide, anticoagulation and quadruple antihypertensive therapy. She died of uncontrollable disease and massive left middle cerebral arterial infarction following an embolism of a left ventricular thrombus and multi-organ failure 12 days after diagnosis. The treatment regimens of both children are shown in Fig. 2.

### Prediction

No additional prognostic factors of adverse outcome were identified using logistic regression. None of the putative predictive variables were independently associated with an increased risk for adverse outcome; therefore, none were able to identify high-risk children early on.

### Secondary outcomes

At last follow-up at a median of 2.1 years, eight of 20 surviving patients (40%) with active disease at diagnosis had continued active disease, while 12 of 20 (60%) were considered inactive. All 20 surviving patients remained on immunosuppressive treatment at last follow-up visit. Median PVAS was 0 (IQR 0–0) and median ITAS2010 0 (IQR 0–1) at last follow-up visit. Of the eight children with ongoing disease activity, two received biologic agents and six non-biologic immunosuppressive therapies. Most of the patients on non-biologic drugs were diagnosed earlier in the study period, prior to first available publications of successful treatment of childhood TAK with biologic agents [10] (Fig. 2). Importantly, 9/12 children with inactive disease on treatment had received biologics; three were treated with non-biologic therapies including corticosteroids, MTX and azathioprine (*p* = 0.02, OR 21.00, 95% CI 1.78–248.30). The five children with biochemically inactive disease at diagnosis maintained inactive disease without treatment throughout the study period until last follow-up.

The most commonly accrued disease damage at last follow-up were vessel stenosis (100% of surviving children), absent pulses (70%), claudication of the extremities (33%), cerebrovascular accidents (26%) and seizures (11%). Median PVDI was 4 (IQR 3–6) and did not significantly differ in the four treatment groups.

## Discussion

This study analyzed one of the largest contemporary childhood TAK cohorts to date. The clinical presentation of childhood TAK was heterogeneous. Overall, 81% of children presented with signs and symptoms of systemic inflammation, active vessel wall disease and critical organ perfusion. Vascular involvement was highly variable; the abdominal aorta and the renal arteries were most commonly affected. Childhood TAK has a dramatic morbidity and early mortality; 48% experienced disease flares and two children died within months of diagnosis. The comparative analysis of treatments revealed that children treated with biologic therapies had significantly fewer flares and achieved inactive disease status more frequently suggesting a potential benefit of biologic agents over non-biologic immunosuppressive drugs.

Children with TAK presented with highly variable clinical phenotypes reflecting the variable location and extent of vascular inflammation. The most common clinical findings at presentation were constitutional symptoms, headaches, blood pressure discrepancy, decreased peripheral pulses and hypertension corresponding to data from published pediatric reports [7, 8, 24]. The majority presented with increased inflammatory markers including CRP and ESR. While the sensitivity of these markers is high for active TAK, they lack specificity [3, 25]. The disease heterogeneity reflects our inability to identify childhood TAK patients at risk for treatment failure and disease flare, which remain a serious concern. We documented a flare rate of 48%, consistent with recent pediatric series (37–4%) [6–8]. Early mortality rate was high (7%); however, mortality rates as high as 27% have been reported in childhood TAK [6, 26, 27].

Children treated with biologic agents had significantly better outcomes compared to children treated with non-biologic therapies. Flare-free survival rates were higher for biologic therapies (TNF-α inhibitors or tocilizumab) compared to non-biologic therapies (80% versus 43% at 2 years, *p* = 0.03). Further, children receiving biologic therapies were more likely to achieve inactive disease at last follow-up compared to children treated with non-biologic agents (MTX, azathioprine, MMF) (*p* = 0.02). Overall, the medications used had reasonable safety profiles over the short observation period. In the pathogenesis of TAK, TNF-α and interleukin-6 (IL-6) were shown to play an important role in promoting vascular inflammation [28, 29]. The effectiveness of TNF-α inhibitors was reported in one pediatric [10] and several adult TAK case series [12, 17, 30, 31]. Mekinian recently found significantly better flare-free survival of adult TAK patients refractory to non-biologic agents when receiving biologic treatments [17].

Biologic therapies have been used in individual patients included in recent pediatric series, but their efficacy has not been systematically evaluated. Filocamo reported four children started on TNF-α inhibitors, either for refractory disease or as first-line agent [10]. Two children achieved remission; the two others had a partial response [10]. The data on TNF-α inhibition in TAK are encouraging, as their role has remained unclear for a long time. Contradictory results have originated from reports of patients, who developed TAK, while treated with TNF-α inhibitors for other diseases [32, 33]. Interestingly, two of our patients were diagnosed with childhood TAK, while being treated with a TNF-α inhibitor for inflammatory bowel disease. Both children responded well to high-dose corticosteroids, the increase of the biologic agent dosing and the addition of MTX. A common genetic background between TAK and ulcerative colitis has recently been suggested with regard to the high rate of co-occurrence of these two diseases [34, 35].

Therapy with the IL-6 inhibitor tocilizumab seems promising; steroid-sparing effects with good clinical and laboratory responses have been described in both pediatric [36–38] and adult TAK series [12, 17, 39–41]. Mekinian documented equivalent efficacy and safety of TNF-α inhibitors and tocilizumab in adult TAK patients refractory to non-biologic therapies [17]. In childhood TAK, tocilizumab has been reported to be effective and well tolerated in a total of 11 TAK children refractory to corticosteroids and non-biologic immunosuppressives [36–38].

In 2008, EULAR published treatment recommendations for large vessel vasculitis in adults [42]. These include the use of high-dose corticosteroids for induction of remission and the consideration of an immunosuppressive agent such as MTX or azathioprine. These are evidence level three recommendations from descriptive studies; data from clinical trials are not yet available. To date, there are no recommendations for childhood TAK. Clearly, further studies including multicenter collaborations are needed to prospectively evaluate the potential benefit of biologic agents in childhood TAK. Until more evidence is available, our data and the recent reports suggest the consideration of biologic agents in childhood TAK patients with critical organ perfusion or end organ damage at diagnosis and in those with severe, treatment refractory disease course.

This study has several limitations. The sample size was small. However, this cohort represents one of the largest worldwide. This retrospective study is spanning three decades during which diagnostic and therapeutic management has evolved. As there is no standardized treatment protocol for TAK, various therapeutic regimens have been used by different pediatric rheumatologists throughout the study period, but interestingly, disease activity measures did not differ between the various treatment groups (Fig. 3). Statistical bias-reducing strategies such as propensity score analysis were not feasible due to the small numbers in the individual groups. Because of small patient numbers, we were also not able to adjust for differences in imaging evaluation, concomitant treatment and other potential confounders of pre- and post-biologic era in the statistical analysis. The PVAS was validated, but may not be the optimal disease activity measurement tool for childhood TAK [21]. However, there is no universally accepted disease activity measure for childhood TAK to date. Finally, our patients were cared for at a tertiary referral hospital and may represent the more severe end of the disease spectrum.

## Conclusions

This contemporary, large childhood TAK cohort study demonstrates the significant disease burden with more than 50% of affected children experiencing adverse outcomes including disease flares or death. The results of this study provide important information regarding treatment efficacy and safety in childhood TAK. Biologic therapies including TNF inhibitors and anti-IL-6 agents resulted in significantly higher 2-year flare-free survival and higher rates of inactive disease at last follow-up compared to non-biologic therapies. Based on this information, current treatment approaches to childhood TAK may need to be revised.

## Abbreviations

ANA: Antinuclear antibody
ANCA: Antineutrophil cytoplasmic antibody
AZA: Azathioprine
CRP: C-reactive protein
CS: Corticosteroids
CTA: Computed tomography angiography
Cyclo: Cyclophosphamide
ESR: Erythrocyte sedimentation rate
IFX: Infliximab
IL-6: Interleukin-6
ITAS2010: Indian Takayasu Arteritis Activity Score
MMF: Mycophenolate mofetil
MRA: Magnetic resonance angiography
MTX: Methotrexate
PVAS: Pediatric Vasculitis Disease Activity Score
PVDI: Pediatric Vasculitis Damage Index
TAK: Takayasu arteritis
TCZ: Tocilizumab
TIA: Transient ischemic attack
TNF-α: Tumor necrosis factor alpha
vWF antigen: von Willebrand factor antigen
